# Cytotoxic Effect of *Rosmarinus officinalis* Extract on Glioblastoma and Rhabdomyosarcoma Cell Lines

**DOI:** 10.3390/molecules27196348

**Published:** 2022-09-26

**Authors:** Eleni Kakouri, Olti Nikola, Charalabos Kanakis, Kyriaki Hatziagapiou, George I. Lambrou, Panayiotis Trigas, Christina Kanaka-Gantenbein, Petros A. Tarantilis

**Affiliations:** 1 Laboratory of Chemistry, Department of Food Science & Human Nutrition, School of Food and Nutritional Sciences, Agricultural University of Athens, Iera Odos 75, 11855 Athens, Greece; 2 Choremeio Research Laboratory, First Department of Pediatrics, National and Kapodistrian University of Athens, Thivon & Levadias 8, 11527 Athens, Greece; 3 Physiotherapy Department, Faculty of Health and Care Sciences, State University of West Attica, Agiou Spiridonos 28, 12243 Athens, Greece; 4 Laboratory of Systematic Botany, Department of Crop Science, School of Plant Sciences, Agricultural University of Athens, Iera Odos 75, 11855 Athens, Greece

**Keywords:** *Rosmarinus officinalis*, phenolic compounds, chemical analysis, glioblastoma, rhabdomyosarcoma, cancer

## Abstract

*Rosmarinus officinalis* is a well-studied plant, known for its therapeutic properties. However, its biological activity against several diseases is not known in detail. The aim of this study is to present new data regarding the cytotoxic activity of a hydroethanolic extract of *Rosmarinus officinalis* on glioblastoma (A172) and rhabdomyosarcoma (TE671) cancer cell lines. The chemical composition of the extract is evaluated using liquid chromatography combined with time-of-flight mass spectrometry, alongside its total phenolic content and antioxidant activity. The extract showed a promising time- and dose-dependent cytotoxic activity against both cell lines. The lowest IC_50_ values for both cell lines were calculated at 72 h after treatment and correspond to 0.249 ± 1.09 mg/mL for TE671 cell line and 0.577 ± 0.98 mg/mL for A172 cell line. The extract presented high phenolic content, equal to 35.65 ± 0.03 mg GAE/g of dry material as well as a strong antioxidant activity. The IC_50_ values for the antioxidant assays were estimated at 12.8 ± 2.7 μg/mL (DPPH assay) and 6.98 ± 1.9 μg/mL (ABTS assay). The compound detected in abundance was carnosol, a phenolic diterpene, followed by the polyphenol rosmarinic acid, while the presence of phenolic compounds such as rhamnetin glucoside, hesperidin, cirsimaritin was notable. These preliminary results suggest that *R. officinalis* is a potential, alternative source of bioactive compounds to further examine for abilities against glioblastoma and rhabdomyosarcoma.

## 1. Introduction

*Rosmarinus officinalis* L. (Lamiaceae), commonly known as rosemary, is a much-branched, evergreen small shrub, usually 50–100 cm tall. It is native in the Mediterranean region and widely cultivated elsewhere for its essential oil, as well as ornamental purposes. Most Greek populations are probably naturalized and originated from cultivated plants, but at least some populations are considered native in the country [1].

Rosemary is considered a typical spice of the Mediterranean diet and it has been characterized as a functional ingredient [2,3]. Traditionally, rosemary leaves have been used against muscle, joint and rheumatism pain [4], as a stimulant and diaphoretic and for its flatulence-relieving properties [5,6]. Headaches, epilepsy, dysmennorhea, inflammation and spasmolytic conditions were also treated with rosemary [7,8]. Nowadays rosemary is among the most studied medicinal plants and its essential oil and extracts’ therapeutic activity has been evaluated against various diseases [9,10]. In particular, *R. officinalis* extracts have been studied for their antioxidant, anticancer, anti-inflammatory, and antimicrobial activity. Ameliorating the status of metabolic and central nervous system (CNS) disorders has also been evaluated [11,12,13,14].

Glioblastoma is an aggressive, malignant cancer of the CNS that originates from the glial cells, characterized by poor survival rate. One reason may be an intrinsic or acquired resistance to radiation and chemotherapy, as many brain tumors could intrinsically manifest a multidrug resistance (MDR) phenotype, thus resulting in relapses or disease progression [15,16]. Rhabdomyosarcoma forms at the soft tissues and more frequently affects the skeletal muscle tissue. It is generally considered a disease of childhood, as most cases are observed between the ages of 0–18 years old. Localized disease is associated with a good prognosis and an overall 5-year survival rate of over 80% with combined surgery, radiation therapy, and chemotherapy. However, in metastatic disease, prognosis is poor with a 5-year event-free survival rate of less than 30% [17,18]. It is the third most common extracranial tumor of the pediatric population, accounting for 4.5% of all cases of childhood cancers.

Both are considered rare types of cancers. Rare-type cancers comprise 22% of the reported cases of cancer [19]. Generally, among the difficulties that accompany a rare disease is the reluctance of pharmaceutical industries to invest time and, of course, a considerable amount of money for the development of a specific therapeutic treatment, since it will be addressed only to a small population. Therefore, one of the ongoing challenges is the continuous gaining of experimental data that will significantly contribute and facilitate the design of specific pharmaceutical formulations. However, independent of the cost required for the research of new pharmacologically active compounds, it should be taken under consideration that many cancer cells are resistant to current therapy due to mutations. Although current therapeutic approaches aim to alleviate symptoms, increase life expectancy and maintain the progression of the disease in remission, they are not few the cases of synthetic formulations leading to severe side effects that are not associated to the disease itself. Consequently, because of limitations regarding the many side effects that impair quality of daily life, cancer drug resistance, rapid increase in the percentage of cancer mortality and numerous new cases diagnosed, scientists are driven towards the development of new therapeutic agents, with fewer or no side effects, to be used as monotherapy or together with current available treatment. To this end, natural products and, in particular, those found in abundance in nature or are easy to cultivate, consist a new area of research, since most of the times the cost of the raw material is affordable and the side effects are usually minimized [20,21].

Given the acquired knowledge from Traditional medicine and the continuous interest in *R. officinalis* as a potential therapeutic agent, the present study aims to evaluate the cytotoxic effect of a hydroethanolic extract of *R. officinalis* against A172 glioblastoma and TE671 rhabdomyosarcoma cell lines, since its effect against these two cancer types has not been previously reported. The biologic activity of a plant is attributed to its chemical profile. However, the chemical profile is strongly dependent on many parameters [22,23]. Therefore, although the chemical characterization of *R. officinalis* extracts has already been given in previous studies [24,25,26], here, we present again the chemistry of the extract used, alongside its total phenolic content and its antioxidant activity.

## 2. Results

### 2.1. Total Phenolic Content and Antioxidant Activity

The extract contains a considerable number of phenolic compounds that corresponds to 35.65 ± 0.03 GAE/g. The extract also exhibited a notable antioxidant activity. The IC_50_ value calculated for the DPPH assay was 12.8 ± 2.7 μg/mL while for the ABTS assay the IC_50_ value was estimated at 6.98 ± 1.9 μg/mL.

### 2.2. Identification of Secondary Metabolites by LC/Q-TOF/HRMS Analysis

Although the chemistry of *Rosmarinus officinalis* is known, we report again its chemical profile, since not only does it depends upon the area, season, and extraction method but it also is essential for explaining its biological activity on A172 and TE671 cell lines.

Characterization of the compounds presented in *R. officinalis* extract was performed with the LC/Q-TOF/HRMS analysis. Most of the compounds identified were flavonoids and phenolic terpenes. Data obtained from the ESI (+) and the ESI (−) ionization mode are summarized in Table 1. Information regarding the generated ms/ms fragmentation process is given as Supplementary Materials. Identification of the compounds detected was based on data obtained from the MassHunter Workstation Software and literature data [26,27,28,29,30].

### 2.3. Evaluation of Cytotoxicity

**Figure 1:**
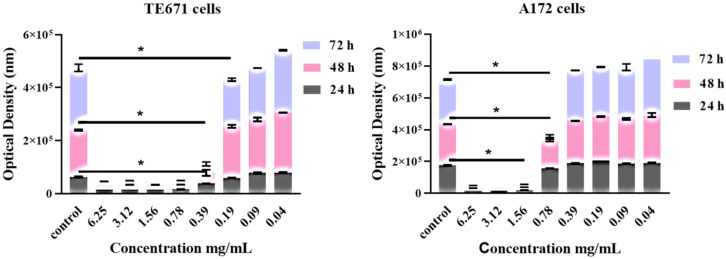
Dose-dependent and time-dependent effect of *R. officinalis* extract on TE671 (**A**) and A172 (**B**) cells. Data are presented as the mean ± standard error of the mean (SEM) (*n* = 8). The asterisk (*) indicates significant differences between untreated and treated cells. The grey color corresponds to 24 h of treatment, the pink to 48 h and the light blue to 72 h.

Both cell lines were exposed to increased concentrations of the extract ranging from 6.25–0.04 mg/mL. The extract exhibited its cytotoxic effect in a dose- and time-dependent manner. Significant differences were observed between the control group and the treated cells, quite at the same range of concentrations. For TE671 cells, the range of the concentrations that reduces cell growth and proliferation ranged from 6.25 mg/mL to 0.39 mg/mL. Note that at the concentration of 0.19 mg/mL, no significant differences were observed at 24 and 48 h of treatment, where proliferation seems to begin. On the contrary, this effect was not observed at 72 h (Figure 1A).

For the A172 cell line, this effect was evident at the concentrations from 6.25 mg/mL to 0.78 mg/mL while at the concentration of 0.39 mg/mL, no statistically significant differences were observed in comparison to the control group, while proliferation of cancer cells had begun.

Interestingly, regarding dose-dependent results and the concentration of 0.78 mg/mL, the effect of the extract was maximal at 72 h after treatment (Figure 1B).

In addition, common for both cell lines is the fact that, for TE671 cells and for the concentrations ranging from 6.25–0.39 mg/mL, the degree of the cytotoxic effect of the extract was the same. For A172 cells, the same was observed for the concentrations ranging from 6.25–1.56 mg/mL. Furthermore, when cells were treated with 0.78 mg/mL and at 24 and 48 h, although the extracts’ cytotoxic activity was still evident, at the same time proliferation of cells had begun slightly. On the contrary, at 72 h of treatment, cancer-cells’ viability had not considerably increased with respect to that of 24 and 48 h.

**Figure 2:**
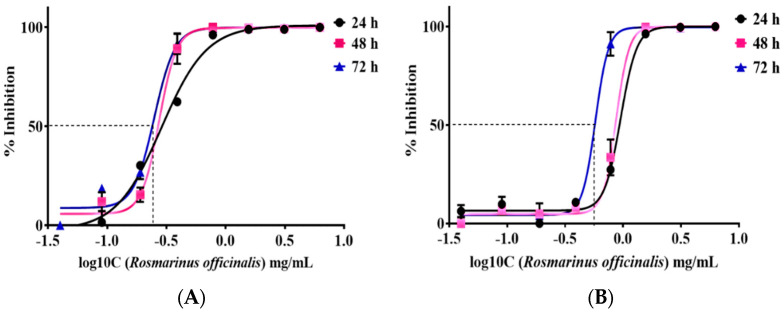
IC_50_ of *R. officinalis* extract on TE671 cells (**A**) and A172 cells at 24, 48 and 72 h (**B**). The lowest IC_50_ value for TE671 cell line was 0.249 ± 1.09 mg/mL, calculated at 72 h and 0.577 ± 0.98 mg/mL for A172 cell line, calculated at 72 h. Cancer -cell viability increases as concentration of the drug decreases.

IC_50_ value, thus, half of the maximal concentration of the tested extract required to inhibit growth and proliferation of cancer cells, was estimated. Dose–response curves regarding all the time points were constructed using a four-parameter logistic model. Normalized results are presented as log10 concentration in Figure 2A for the TE671 cancer cell line and in Figure 2B for the A172 cancer cell line. In the case of TE671 cells, the lowest IC_50_ value was estimated at 0.249 ± 1.09 mg/mL at 72 h after treatment with the extract. The IC_50_ values at 24 and 48 h were calculated at 0.287 ± 1.22 mg/mL and 0.274 ± 1.4 mg/mL, respectively. Regarding A172 cells, the lowest IC_50_ value was observed at 0.577 ± 0.98 mg/mL at 72 h after treatment. For the first 24 h, the IC_50_ value was calculated at 0.952 ± 1.11 mg/mL and after 48 h of treatment the corresponding value was found to be 0.871 ± 1.36 mg/mL. IC_50_ values decreased with increasing exposure time. The calculated values demonstrated that TE671 cells are more sensitive to the extract, since the IC_50_ value is lower than that of A172 cells. In addition, as is presented in Figure 2, the behavior of TE671 cells at all time points is almost the same, given the fact that IC_50_s do not differ considerably. On the contrary, for A172 cells, those values are rather close for the first 24 and 48 h; however, at 72 h, IC_50_ significantly decreases. This might be attributed to the population doubling time, which reached 80 h for TE671 cells and 40 h for A172 cells.

**Figure 3:**
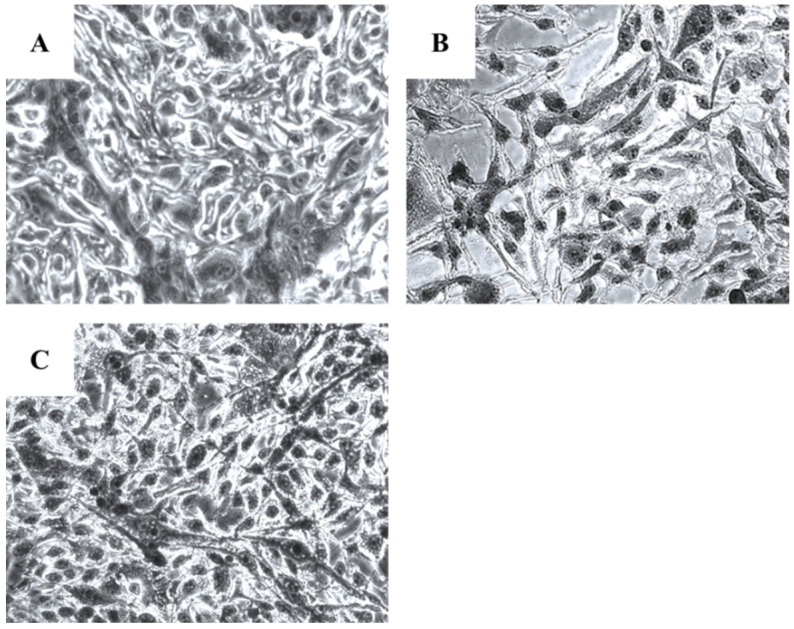
Microscopic images of the TE671 rhabdomyosarcoma cells, grown for 72 h in DMEM with no other treatment (**A**), cells treated with 0.39 mg/mL of the extract (**B**) and cells treated with 0.19 mg/mL of the extract (**C**). Images were captured at ×200 magnification.

**Figure 4:**
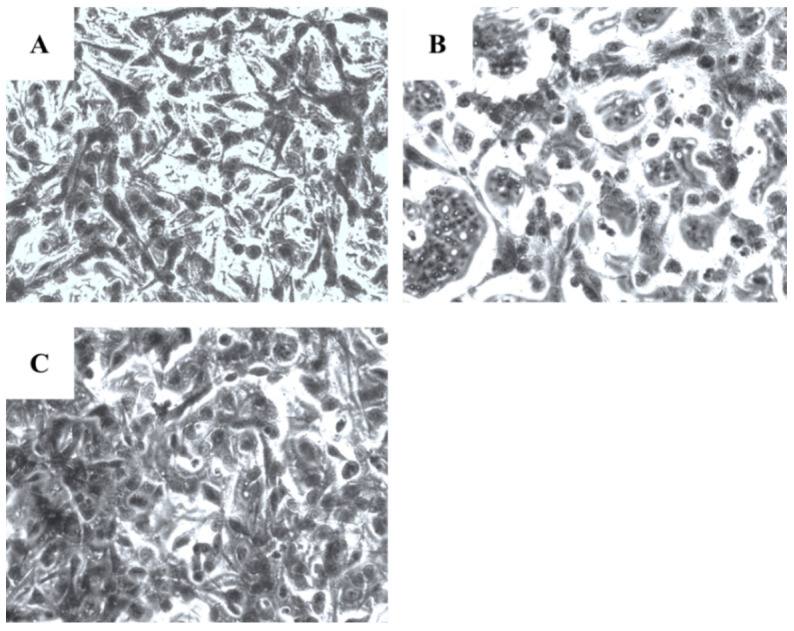
Microscopic images of the A172 glioblastoma cells, grown for 72 h in DMEM with no other treatment (**A**), cells treated with 0.78 mg/mL of the extract (**B**) and cells treated with 0.39 mg/mL of the extract (**C**). Images were captured at ×200 magnification.

Microscopical investigation of TE671 is presented in Figure 3A–C. More precisely, in Figure 3A, cells are confluent since they have undergone any treatment, while at the concentration of 0.39 mg/mL cells are significantly reduced (Figure 3B). In Figure 3C, which corresponds to the concentration of 0.19 mg/mL, cells proliferation has begun. In the case of A172 cells, the same behavior was observed (Figure 4A–C). Figure 4A represents those cells that have received no treatment. At the concentration of 0.78 mg/mL, cells are less confluent (Figure 4B), while at the concentration of 0.39 mg/mL, proliferation of cells is evident (Figure 4C).

## 3. Discussion

*R. officinalis* is a plant known for its potent antioxidant activity as it has been evaluated in many studies and with different antioxidant assays. Such activity is mainly attributed to the presence of rosmarinic acid, carnosic acid, carnosol and rosmanol [25,31,32]. It has been proposed that the catechol group of these compounds is responsible for their antioxidant activity [25].

*R. officinalis* belongs to the Lamiaceae family, a well-known family which includes a variety of plant species that contain a plethora of bioactive compounds [33]. *R. officinalis* leaves’ extracts have been studied for their chemical composition and the presence of multiple compounds that belong to flavonoids (apigenin, genkwanin, scutellarein), phenolic diterpenes (carnosol, rosmanol, epirosmanol, carnosic acid), triterpenes (ursolic acid, betulinic acid) and caffeic acid esters (rosmarinic acid) has been reported [24,25].

Results reported in previous studies are in accordance with data presented here. In particular, in our study, chemical analysis of the hydroethanolic extract of *R. officinalis* showed the presence of rosmarinic acid, hydroxycinnamic acids, flavonoids and phenolic terpenes. According to the relative abundance as generated by the MassHunter software, carnosol was the compound presented in abundance followed by rosmarinic acid. Rosmanol, epirosmanol and rosmaridiphenol are metabolites derived from carnosic acid. Carnosol is an oxidized derivate of carnosic acid, produced via a non-enzymatic reaction [34]. References do report both the presence of carnosic acid and carnosol in *R.officinalis* plants [29,30,35]. Furthermore, many studies indicate that plants of the genus *Rosmarinus* grown in the Mediterranean, area are a very rich source of carnosic acid [25,36,37]. However, under extreme environmental conditions and in order for the plant to protect itself from various exogenous invasions, oxidative stress is unavoidable. That means that abiotic-induced stress was possibly the main reason for the oxidation of carnosic acid to carnosol, as well as for the presence of other oxidation metabolites, as previously reported [34,36].

Three are the most studied compounds isolated from *R. officinalis* extracts, carnosic acid, carnosol and rosmarinic acid. Carnosic acid is a compound commonly found in Lamiaceae species and was first isolated from *Salvia officinalis* [38]. Later, it was also found in abundance in rosemary which is yet considered as the richest source of all the Lamiaceae family plants. Chemically, carnosic acid is a phenolic diterpene and has been studied for its health-promoting properties, namely, antioxidant, antitumor, chemo-preventive, anti-inflammatory and hypoglycemic [39]. Carnosol belongs to phenolic diterpenes. It is a strong antioxidant, anticancer, chemo-preventive and anti-inflammatory agent [12,34,40]. The third well-studied compound of the plant is rosmarinic acid. Rosmarinic acid has been documented as a strong antioxidant and antimicrobial compound and it has also been tested against different cancer cell lines and against skin-irritating conditions such as atopic dermatitis [41,42,43,44]. Although the above three compounds are usually found in abundance in *R. officinalis* extracts, the therapeutic activity of the plant specifically regarding cancer, is not attributed only to these [45]. It has been observed that extracts from *R. officinalis* exert better antitumor activity with respect to its isolated compounds, precisely carnosol, ursolic and carnosic acid [46,47]. Interestingly, in the study of González-Vallinas et al., (2014) [47], a combination of carnosic acid and carnosol presented a better antiproliferative activity probably due to the synergistic effect of the two compounds. Given the cost advantages for a pharmaceutical company regarding the use of an extract rather than purified compounds and taking into account the above-mentioned findings, in this study a hydroethanolic extract derived from the leaves of the plant was used, to evaluate its cytotoxic activity against A172 and TE671 cancer cell lines. According to our knowledge, this is the first time that the cytotoxicity of *R. officinalis* has been evaluated against these two specific cell lines.

In our study, we observed that treatment with *R. officinalis* manifested a threshold-like mechanism, as it appeared that up to certain concentrations the extract manifested similar toxicity as the control sample and, on the other hand, after a certain “step” (0.39 mg/mL) the extract becomes effective. This phenomenon was not only dose-dependent but also time-dependent, as it manifested the same behavior at 24 h, 48 h and 72 h. This type of action is reported for the first time. Studies concerning the effects of *R.officinalis* on prostate cancer cells [48,49], melanoma [50] and in hematopoietic, epithelial, and mesenchymal tumor cell types [51] manifested a gradual dose-dependent type of action. Thus, the most interesting conclusion from these observations is that *R. officinalis* acts on tumor cell survival differently, depending on the cell type. All studies agree that the extract is effective against tumor cells, yet the fact that it acts in a cell-dependent manner urges towards a more in-depth investigation into its mechanics.

*R. officinalis* anticancer effects against glioblastoma cell lines have been previously described. U87MG has been used and it was shown that an aqueous extract of the plant (1/75 *v*/*v* dilution) managed to inhibit cancer cell proliferation by 42%. On the contrary, rosemary extract boosted the viability of mouse embryonic fibroblasts cells (MEF) by 9.5%. Authors compared the efficacy of the extract with that of etoposide, a highly toxic agent that causes myelosuppression. Etoposide reduced cell viability to a higher degree with respect to *R. officinalis*. However, authors also showed that co-treatment with the extract and etoposide does not influence the chemotherapeutic agent toxicity but increases cells rate inhibition. Nevertheless, rosemary extract does not seem to inhibit growth in MEF cells to the same degree as etoposide [52].

Carnosol was examined for its potent cytotoxic activity on the U87MG glioblastoma cell line. Using a range of concentrations between 100 nM–60 μM, carnosol not only significantly inhibited in a dose-dependent manner cancer cell viability at 48 and 72 h of treatment, but also its anti-proliferative effect continued even after washing the substance. Furthermore, the compound did not promote the metastasis of cancer cells. The same effect was also observed when cells were treated with a mixture of carnosol and temozolomide, an alkylating agent, used to treat brain tumors. In addition to this, carnosol potentiated the cytotoxic effect of temozolomide. Of note, also, is that carnosol did not affect the proliferation of healthy cells. In addition, since U87MG cell lines express the p53 gene, the possible involvement of carnosol in the p53-activation pathway was investigated. A re-activation of p53 and the concomitant activation of BAX protein and deactivation of Bcl-2 were observed [53]. The results of our study are in accordance with the above-mentioned studies. The extract of *R. officinalis* inhibited the growth and proliferation of A172 glioblastoma cells. In contrast to the study of Giacomelli et al. (2016) [53], in our study, the extract exhibited its antiproliferative effect after 24 h of treatment and the peak of its effect was observed at 72 h. Nonetheless, its cytotoxic potency did not outweigh that of carnosol expressed as IC_50_ values.

Chemotherapeutic treatment of rhabdomyosarcoma includes the use of agents such as doxorubicin, vinblastine, and etoposide. Combination of these drugs with rosemary extracts allowed to diminish the concentration of the chemical agent, thus reducing its toxic effects [45,54]. Regarding the cytotoxic activity of *R. officinalis* on rhabdomyosarcoma cell lines, there is lack of literature data. According to our results, the extract used is capable of inhibiting cancer cell proliferation by exerting its best activity at 72 h after treatment.

In general, many studies report the use of secondary metabolites against cancer [55,56,57,58,59,60].

Regarding the family of phenolic compounds, those belong to the most studied biomolecules. The anticancer activity of phenolic compounds has been demonstrated in a variety of malignant cell lines such as HT-1080 fibrosarcoma cell line, HT-44 melanoma cells, HT-20, HT-29 and DLD-1 colon cancer cells, MCF-7, MDA-MB 468 and 231, T47D breast cancer cells, PC-3 and LNC prostate cancer cells, HS-22 lung cancer cells, SGC-7901 gastic cancer cells, cervical cancer cells (HeLa), human leukemia (HL-60) and NB-4 promyelocytic leukemia cells, adenocarcinomic human alveolar basal epithelial cells (A549) and OAW adenocarcinoma cancer cells [58,61].

In our study, visual observation of microscopic images of the cells demonstrated that the extract exerts its cytotoxic activity by reduction in cell population. In addition, reduction in cell size was observable, as well as a nuclei fragmentation, which confirmed the observed cytotoxicity through the photometric method. This observation gave us a hint for the type of cell death caused by the extract, yet with more investigations in need to confirm.

A lot of mechanisms have been proposed to explain the cytotoxic effect of phenolic compounds. For example, phenolics chemoprotective/ anticancer activity is mainly due to their antioxidant and anti-inflammatory properties and many studies relate a phenolic rich diet with minor incidence of cancer development [62,63,64,65]. Many researchers have pointed out the potential of these molecules to interfere with crucial signaling pathways of the proliferation, migration, differentiation, apoptosis and angiogenesis of cancer cells [61,66,67,68]. For example, cinnamic and benzoic acid induce their antiproliferative effect on melanoma and breast cancer cells by interrupting the S and G2/M phase, respectively. Furthermore, caffeic acid, 5-caffeoylquinic acid, di-caffeoylquinic acid, ferulin and p-coumaric acid exert a potent antiproliferative effect against various cancer cell types [61]. In addition, cell death is another point that has been evaluated using phenolic compounds. Arrest of the cell cycle at Go/G1 phage, morphological changes in cancer cells; activation of apoptosis regulators such as caspaces and Bax protein and p53 and p21 genes; downregulation of transcription factors, such as transcription factor kappa B (NF-kB) and Bcl-2 (B-cell lymphoma 2) gene; and inhibition of enzymes vital for DNA transcription are some examples that confirm the potential of phenolic compounds to accelerate cancer cell death [63,69].

## 4. Materials and Methods

### 4.1. Plant Material

Plant material of *R. officinalis* was collected from the Botanical Garden of Philodassiki Enossi Athinon, at the foothills of Mt Hymettus (Attica, Greece). The living collection established in the Botanical Garden originated from a native population located in Ritsona area (eastern Sterea Ellas, Greece). Voucher specimen was deposited at the Herbarium of the Agricultural University of Athens (ACA), with the following label: Greece, Sterea Ellas, prefecture of Attiki, Botanical Garden of Philodassiki Enossi Athinon, alt. 360 m, 37°57′ N, 23°47′ E, 20.09.2016, Trigas 6327, ACA.

### 4.2. Sampling Extraction

Four grams of dried *R. officinalis* leaves were extracted as previously described by Kakouri et al., (2019) [70], in an ultrasonic water bath using a hydroethanolic solution (70% *v*/*v*). Extraction took place in triplicate.

### 4.3. Total Phenolic Content and Antioxidant Activity

Total phenolic content was performed using Folin–Ciocalteu reagent (0.2N) and gallic acid to construct the calibration curve. The experiment took place as previously described by Kakouri et al., (2019) [70]. Results were expressed as mg of gallic acid equivalents (GAE) per gram of dry material, derived from threefold measurements and according to the following equation:

y = 0.0012x + 0.012 (r = 0.998)(1)

The antioxidant activity was estimated using the 2,2-Diphenyl-1-picrylhydrazyl (DPPH•) and the 2,2′-azinobis [3-ethylbenzthiazoline-6-acid] (ABTS•+) radical scavenging assays. The experimental procedure followed that of Kakouri et al., 2019 [70]. For both the assays trolox was used as standard antioxidant. Results were expressed as IC_50_ values and according to the following equation:

% Inhibition = (Acontrol − Asample)/Acontrol × 100(2)

### 4.4. LC/Q-TOF/HRMS Conditions

To identify the chemical profile of *R. officinalis* extract an HPLC system (high performance liquid chromatography) consisting of a degasser, autosampler, quaternary pump, diode array detector, and column oven (Agilent Series 1260, Agilent Technologies, Santa Clara, CA, USA), coupled to a 6530 Q-TOF mass spectrometer (Agilent Technologies, Santa Clara, CA, USA) was used. Experimental conditions were adjusted as in the study of Kakouri et al., (2019) [70]. The extract was analyzed under the positive and negative ionization mode. The parameters set for the Q-TOF mass analysis follow those described at our previous analysis [70]. CID-ms/ms spectra were recorded on the auto MS/MS mode. Mass range was set to 50–1000 and collision energy was set at 40 V. Results were analyzed using the Agilent MassHunter Workstation software LC-MS Data Acquisition for 6530 series Q-TOF (version B07.00, Agilent Technologies, Santa Clara, CA, USA).

### 4.5. Evaluation of Cytotoxicity

#### 4.5.1. Cells Treatment before and after Exposure to the Extract

The TE671 rhabdomyosarcoma cancer cell line was obtained from the European Collection of cell cultures (ECACC, London, UK). The A172 glioblastoma cell line was obtained from a male patient of 53 years old (ECACC, London, UK, Cat. Nr 88062428). Cells were grown in a cell-culture flask (75 cm^2^ surface area) in Dulbecco’s modified Eagle’s Medium (DMEM) (ThermoFisher Scientific Inc. (Gibco), Waltham, MA, USA Cat. Nr. 10566016) enriched with glucose (4500 mg/mL), and 15% fetal bovine serum (FBS) (ThermoFisher Scientific Inc. (Gibco), Waltham, MA, USA Cat. Nr. 26140-079), L-glutamine (2 mM) (ThermoFisher Scientific Inc. (Gibco), Waltham, MA, USA Cat. Nr. A12860-01). A dual antibiotic solution of penicillin G (100IU) and streptomycin (100 μg/mL) was added (ThermoFisher Scientific Inc. (Gibco), Waltham, MA, USA Cat. Nr. 15140-122), in addition to an amphotericin B solution (ThermoFisher Scientific Inc. (Gibco), Waltham, MA, USA Cat. Nr. 14140-122). A Coulter counter apparatus was used to measure the number of cells inoculated in the experimental setup. Cells were seeded in 96-well plates (1.5 × 10^3^ cells/mL) and were allowed to grow for 24 h until reaching ~80% confluence. After 24 h, cells were exposed to successive diluted concentrations of the extract (t = 0 h), ranging from 0.04 to 6.25 mg/mL, derived from the dried extract diluted de novo in DMSO (10% *v*/*v*). The final concentration of DMSO when the extract was added to cell culture was 1% *v*/*v*. A control well with the same concentration of DMSO was used to confirm that no toxic effect was observed. Cells were incubated for 24, 48 and 72 h.

Experiments were performed in 96-well plates (CellStar^®^ Sigma-Aldrich Chemie GmbH, Taufkirchen, DE Cat. Nr. M3687-60EA, Saint Louis, MO, USA). Plate set up was as follows: a column contained only cell culture medium, a column of cell culture and the staining chemical, a column with cultured cells and a column with cultured cells plus the staining chemical. The remaining wells were used for the testing of the extract in various concentrations. As blank were used those wells containing cell culture medium only, cells and no staining agent or drug, whereas as positive control were used those wells with cultured cells without staining agent. All experiments were performed in triplicate.

#### 4.5.2. Alamar Blue Assay

Assessment of cell viability after incubation with testing agents was performed with resazurin reduction experiments, using Alamar Blue viability assay. Cell viability at each time point (24, 48, 72 h) was quantified by adding Alamar Blue (Gibco, Invitrogen Inc. Carlsbad, CA, USA) to each well. Treated cells were supplemented with 10% alamar blue reagent and incubated for 6 h at 37 °C. Wells that contained only alamar blue were considered as blank while positive control were considered those wells that contained the untreated cells. Percent viability was calculated according to the following formula:

V (%) = (OD1 − OD2 − Blank)/(OD3 − OD2) × 100(3)

where OD1 stands for the optical density in nm for treated with the chemical cells, OD2 stands for the optical density in nm for those well containing nutrient medium and the chemical and OD3 stands for the cells that were not exposed to the chemical. Optical density was read at 570 nm. Results were expressed as IC_50_ values; thus, the concentration of the chemical that causes 50% inhibition with respect to the untreated cells.

#### 4.5.3. Giemsa Staining

Cells were colored with the Giemsa stain. Briefly, 100 μL of pure ethanol were added to a 96-well plate containing the treated cells after removing the nutrient medium. Cells were left in ethanol for five minutes and then 100 μL of the Giemsa stain were added. Plates remained for 15 min at room temperature, followed by stain removal and cell washing with 100 μL of NaCl 0.9% (*v*/*v*). Cells were microscopically observed at 24, 48 and 72 h of incubation.

#### 4.5.4. Data Analysis

The GraphPad Prism (version 8.4.2, GraphPad Software for Windows, San Diego, CA, USA) was used to calculate the IC_50_ value according to a four-parameter logistic model. Dose- and time-dependent effect of the tested extract with respect to the control group were also calculated with GraphPad Prism (version 8.4.2). All data were presented as mean ± standard error of the mean (SEM). Statistical differences between untreated and treated cells were evaluated with the Student *t*-test. *p* values < 0.05 were considered statistically significant and confidence intervals were at ±95% (±95% CI). Normalized results are presented as log10 concentration.

## 5. Conclusions

In conclusion, the extract examined in this study highlights, for the first time, the cytotoxic effect of *R. officinalis* against TE671 and A172 cancer cell lines. The extract inhibited cancer cell proliferation in a dose- and time-dependent manner, with TE671 cells being more susceptible to the treatment with the extract. A further approach of this study would be to determine the mechanism by which *R. officinalis* exhibited its cytotoxic activity. However, our results are a first approach to the use of the plant as a candidate therapeutic agent. As expected, the extract presented notable antioxidant activity, high total phenolic content and a rich chemical profile. Given the importance of phenolic compounds as potent antioxidant molecules and the connection of antioxidant activity with cancer treatment, this Mediterranean plant is certainly worth further investigation.

## Figures and Tables

**Table 1:** Tentatively identified compounds of *Rosmarinus officinalis* leaves at the positive and negative ionization mode.

Peak Number	Identification	Molecular Formula	ESI (+)				ESI (−)			
			Observed Mass	Mass Error (Δ*m*)	[M+H]^+^ (*m*/*z*)	t_R_	Observed Mass	Mass Error (Δ*m*)	[M-H]^−^ (*m*/*z*)	t_R_
1	caffeic acid hexoside	C_15_H_18_O_9_	343.1023	0.00	163.0387; 145.0273; 135.0428	2.32	341.0875	−0.91	179,0340; 161,0237; 135.0442	1.97
2	caffeic acid	C_9_H_8_O_4_	181.0496	0.39	163.0385; 135.0444; 117.0328	2.68	n.d			
3	chlorogenic acid	C_16_H_18_O_9_	355.1023	0.00	163.0385; 145.0264; 135.0424	2.97	353.0873	−1.44	191.0547; 179.0336; 173.0451; 135.0446	2.90
4	tuberonic acid	C_12_H_18_O_4_	227.1278	0.08	209.1138; 191.1068; 163.1114;	3.56	739.1672	0.54	449.0852; 339.0510; 177.0177	4.78
5	rhamnetin hexoside	C_22_H_22_O_12_	479.1181	−0.63	317.0648; 302.0425; 163.0381	7.05	n.d.			
6	hesperidin	C_28_H_34_O_15_	611.1968	−0.41	303.0857; 285.0757; 195.0284; 153.0180	7.81	609.1453	−1.51	300.0268; 271.0241; 255.0292; 151.0032	5.97
7	apigenin glucoside	C_21_H_20_O_10_	433.1129	−0.05	271.0602; 119.0468	7.88	463.0871	−1.51	300.0267; 271.0240; 151.0029	6.45
8	hispidulin rutinoside	C_28_H_32_O_15_	609.1821	1.15	463.1221; 301.0702; 269.0288	8.19	593.1509	−0.50	327.0473; 285.0388; 255.0288; 227.0343; 151.0054	7.06
9	rosmarinic acid hexoside	C_24_H_26_O_13_	n.d				521.1292	−1.67	359.0800; 179.0334; 133.0305	7.32
10	rosmarinic acid	C_18_H_16_O_8_	361.0918	0.28	181.0473; 163.0386; 135.0341	8.25	359.0764	2.34	197.0445; 179.0337; 161.0236	8.30
11	umbelliferone	C_9_H_6_O_3_	163.0391	0.80	145.0279; 117.0331	8.52	n.d			
12	luteolin-acetyl-glucuronide	C_23_H_20_O_13_	n.d				503.0828	−0.64	399.0726; 285.0390; 199.0381; 151.0016; 133.0285	9.56
13	methyl rosmarinic acid	C_19_H_18_O_8_	n.d				393.09220	−1.85	359.0758 373.0922; 179.0341; 135.0442	9.59
14	cirsimaritin hexoside	C_23_H_24_O_11_	477.1395	0.84	300.0861; 282.0507	9.59	n.d			
15	cirsimaritin	C_17_H_14_O_6_	315.0866	0.92	300.0615; 282.0512	13.09	313.0712	−1.79	298.0467; 283.0241	12.99
16	rosmanol	C_20_H_26_O_5_	347.1857	0.29	301.1785; 283.1676	13.83	345.1703	−1.30	301.1791; 283.1691	13.43
17	methyl umbelliferone	C_10_H_8_O_3_	177.0546	−0.11	149.0230; 93.0310	14.45	n.d.			
18	salvigenin	C_18_H_16_O_6_	329.1020	0.12	296.0680; 268.0727	16.85	285.0392	−4.56	267.0258; 213.0525; 151.9210 133.0281	9.14
19	rosmadial	C_20_H_24_O_5_	n.d				343.1544	−2.04	300.0996	17.67
20	epirosmanol methyl ether	C_21_H_28_O_5_	n.d				359.1856	−2.22	329.1742; 283.1695; 285.1781	17.96
21	carnosol	C_20_H_26_O_4_	331.1900	−1.15	285.1844; 243.1364	18.51	329.1748	−3.13	285.1852	18.51
22	carnosol isomer	C_20_H_26_O_4_	331.1902	−0.54	285.1848; 243.1385	18.62	n.d			
23	rosmaridiphenol	C_20_H_28_O_3_	317.2112	0.00	299.1998; 285.1872; 281.1906	19.97	n.d.			

n.d: not detected.

## Data Availability

Not applicable.

## Supplementary Materials

The following supporting information can be downloaded at: https://www.mdpi.com/article/10.3390/molecules27196348/s1, File S1: Detailed description of the ms/ms fragmentation process.

## Sample Availability

Samples of *R. officinalis* extract are available from the authors.
