# Supplementary material for: Cytotoxic Effect of Rosmarinus officinalis Extract on Glioblastoma and Rhabdomyosarcoma Cell Lines
Source: Molecules. 2022 Sep 26;27(19):6348. doi: 10.3390/molecules27196348 (PMC9573533; doi:10.3390/molecules27196348)
Supplement: Supplementary file 1 [file molecules-27-06348-s001.zip › molecules-1917437-supplementary.pdf]

molecules

MDPI

Supplementary material

Cytotoxic Effect of *Rosmarinus officinalis* Extract on Glioblastoma and Rhabdomyosarcoma Cell Lines

Eleni Kakouri [1], Olti Nikola [2], Charalabos Kanakis [1], Kyriaki Hatziagapiou [2,3], George I. Lambrou [2], Panayiotis Trigas [4], Christina Kanaka-Gantenbein [2] and Petros A. Tarantilis [1,*]

[1] Laboratory of Chemistry, Department of Food Science & Human Nutrition, School of Food and Nutritional Sciences, Agricultural University of Athens, Iera Odos 75, 11855 Athens, Greece
[2] Choremeio Research Laboratory, First Department of Pediatrics, National and Kapodistrian University of Athens, Thivon & Levadias 8, 11527 Athens, Greece
[3] Physiotherapy Department, Faculty of Health and Care Sciences, State University of West Attica, Agiou Spiridonos 28, 12243 Athens, Greece
[4] Laboratory of Systematic Botany, Department of Crop Science, School of Plant Sciences, Agricultural University of Athens, Iera Odos 75, 11855 Athens, Greece
* Correspondence: ptara@aua.gr; Tel.: +30-210-529-4262

Detailed Description of the ms/ms Fragmentation Process

The low energy CID MS/MS spectrum of the precursor protonated molecule $[M+H]^+$ at *m/z* 343.1023 (compound **1**) afforded a product ion at *m/z* 181.0501 which indicated the loss of a hexose moiety, revealing simultaneously the presence of caffeic acid which was confirmed by further fragment ions at *m/z* 163.0387, at *m/z* 145.0273 and at *m/z* 135.0428. Compound **1** was identified as caffeic acid hexoside.

Similarly, the low energy CID MS/MS spectrum of the precursor protonated molecule $[M+H]^+$ at *m/z* 181.0496 afforded similar fragment ions, namely, at *m/z* 163.0385, at *m/z* 145.0271, at *m/z* 135.0444, at *m/z* 117.0328. Compound **2** was identified as caffeic acid.

The product ion scan of the precursor protonated molecule $[M+H]^+$ at *m/z* 355.1023 afforded a product ion at *m/z* 163.0385. The loss in 192.0638 Da was attributed to a quinic acid moiety, while the presence of the fragment ion at *m/z* 163.0385 confirmed the presence of caffeic acid. Compound **3** was identified as chlorogenic acid, which was further supported by the presence of product ions at *m/z* 145.0264 and at *m/z* 135.0424.

The product ion scan of the precursor protonated molecule $[M+H]^+$ at *m/z* 227.1278 afforded product ions at *m/z* 209.1138, at *m/z* 191.1068 and at *m/z* 163.1114 formed by consecutive losses of two -OH groups and a -C=O group, respectively. A product ion at *m/z* 149.0949 was obtained derived from the loss of a -CH3 group $[M+H\text{-}163.1114\text{-}CH3]^+$ and at *m/z* 95.0472, was produced by the cleavage of a pentane moiety. According to the molecular formula of Compound **4**, as generated by the MassHunter WorkStation software $[C_{12}H_{18}O_4]$, it was identified as tuberonic acid.

The low energy CID MS/MS spectrum of the precursor protonated molecule $[M+H]^+$ at *m/z* 479.1181 afforded a product ion at *m/z* 317.0648 formed by the loss of a hexose moiety at *m/z* 302.0425 $[M+H\text{-}CH_3]^+$ and at *m/z* 163.0381 formed by the cleavage of the ${}^{0.2}A^+$ ring. According to the molecular formula of compound **5** $[C_{22}H_{22}O_{12}]$, it was identified as rhamnetin hexoside.

The product ion scan of the precursor protonated molecule $[M+H]^+$ at *m/z* 611.1968 afforded a product ion at *m/z* 303.0857 derived from the loss of two sugar moieties, a hexose and a pentose. Further product ions at m/z 285.0757 $[M+H\text{-}308.1111\text{-}H_2O]^+$, at *m/z* 195.0284 $[{}^{0.4}B^+$ cleavage] and at *m/z* 153.0180 $[{}^{1.3}A^+$ cleavage] allowed the identification of Compound **6** as hesperidin.

The low energy CID MS/MS spectrum of the precursor protonated molecule $[M+H]^+$ at *m/z* 433.1129 afforded a product ion at *m/z* 271.0602 derived from the cleavage of a hexose moiety. The derived ion was suggested to belong to apigenin and this was supported

by the presence of the product ion at *m/z* 119.0468 which derived from the $^{1,3}B^+$ ring cleavage. Compound **7** was identified as apigenin glucoside.

The product ion scan of the precursor protonated molecule $[M+H]^+$ at *m/z* 609.1821 afforded a product ion at *m/z* 463.1221 and at *m/z* 301.0702 derived from consecutive losses of a deoxyhexose and a hexose moieties, followed by a product ion at *m/z* 269.0288 produced by the loss of a -CH_3O group. The aglycon part was identified as hispidulin and Compound **8** was tentatively identified as hispidulin rutinoside.

The product ion scan of the precursor protonated molecule $[M+H]^+$ at *m/z* 361.0918 afforded a product ion at *m/z* 181.0473 and at *m/z* 163.0386 $[M+H-181.0473-H_2O]^+$ and at *m/z* 135.0431 derived from cleavage of the 8C-9C bond and giving rise to dihydrocaffeic acid. According to literature data and available authentic standard solution, Compound **10** was identified as rosmarinic acid.

The low energy CID MS/MS spectrum of the precursor protonated molecule $[M+H]^+$ at *m/z* 163.0392 afforded a product ion at *m/z* 145.0279 derived from the loss of a H_2O molecule. Further product ions at *m/z* 117.0331 and at *m/z* 89.039 were produced by the loss of a -CO and a OH group from position 7 of the benzene ring. Compound **11** was identified as umbeliferone.

The low energy CID MS/MS spectrum of the precursor protonated molecule $[M+H]^+$ at *m/z* 477.1395 afforded a product ion at *m/z* 315.0861 derived from the loss of a hexose moiety. Based on further product ions at *m/z* 300.0861 $[M+H\text{-hexose-}CH_3]^+$ and at *m/z* 282.0507 $[M+H\text{-hexose-}H_2O]^+$ and on literature data, Compound **14** was identified as cirsimaritin hexoside.

Similar fragmentation as for compound 14, presented also for compound **15**, regarding the aglycon part. Thus, compound **15** was identified as cirsimaritin.

The product ion scan of the precursor protonated molecule $[M+H]^+$ at *m/z* 347.1855 afforded the following product ions: at *m/z* 301.1785 and at *m/z* 283.1676 derived from the loss of a -CHO_2 and a water molecule, respectively. According to literature data, Compound **16** was identified as rosmanol.

Mass spectra of *Rosmarinus officinalis* L. extract allowed the identification of a compound analogue of umbelliferone. The low energy CID MS/MS spectrum of the precursor protonated molecule $[M+H]^+$ at *m/z* 177.0546 afforded a product ion at *m/z* 149.0230 derived from the loss of a -C_2H_3 group. Another fragment ion at *m/z* 93.0310 produced by the cleavage at position 8C-9C, helped in identifying compound **17** as methylumbelliferone.

Compound **18** was tentatively identified as salvigenin. The low energy CID MS/MS spectrum of the precursor protonated molecule $[M+H]^+$ at *m/z* 329.1020 afforded two product ions at *m/z* 296.0680 and at *m/z* 268.0727 which were formed by consecutive losses of two -CH_3O groups.

The low energy CID MS/MS spectrum of the precursor protonated molecule $[M+H]^+$ at *m/z* 331.1900 afforded a product ion at *m/z* 285.1844 derived from the loss of a -CHO_2 group and a product ion at *m/z* 243.1364 derived from the cleavage of a propene moiety. According to literature data, Compound **21** was identified as carnosol. One more isomer of carnosol was identified (Compound **22**).

The low energy CID MS/MS spectrum of the precursor protonated molecule $[M+H]^+$ at *m/z* 317.2112 afforded a product ion at *m/z* 299.1998 $[M+H-OH]^+$ and at *m/z* 285.1872 [M+H-32.0240] formed by the loss of two methyl groups and at *m/z* 281.1906 $[M+H-H_2O-OH]$. Compound **23** was identified as rosmaridiphenol.

The low energy CID MS/MS spectrum of the precursor deprotonated molecule $[M-H]^-$ at *m/z* 521.1292 afforded a product ion at *m/z* 359.0800 derived from the loss of a hexose moiety. The aglycon part remained bearing the molecular formula $C_{18}H_{15}O_8$ was identified as rosmarinic acid and this was further confirmed by subsequent product ions at *m/z* 179.0334 and at *m/z* 133.0305, which were similar to those of the ESI(+). Compound **9** was identified as rosmarinic acid hexoside.

The low energy CID MS/MS spectrum of the precursor deprotonated molecule $[M-H]^-$ at *m*/*z* 503.0826 afforded a main product ion at *m*/*z* 285.0390 after a minor fragment ion at *m*/*z* 399.0726 formed by the loss of an acetate and a carboxylic acid group. Further minor fragment ions at *m*/*z* 199.0381, at *m*/*z* 151.0016 and at *m*/*z* 133.0285 formed by the loss of C_2H_2O and CO_2 groups and cleavage of the $^{1.3}A^-$ and $^{1.3}B^-$ rings, respectively. Compound **12** was identified as luteolin acetyl glucuronide.

The product ion scan of the precursor deprotonated molecule $[M-H]^-$ at *m*/*z* 373.0922 presented the molecular formula $C_{19}H_{18}O_8$. Fragmentation pathway was similar to that of rosmarinic acid; consequently, Compound **13** was identified as methyl rosmarinic acid.

The low energy CID MS/MS spectrum of the precursor deprotonated molecule $[M-H]^-$ at *m*/*z* 343.1544 afforded a main product ion at *m*/*z* 300.0996 formed by the loss of three methyl groups. Compound **19** was identified as rosmadial.

The product ion scan of the precursor deprotonated molecule $[M+H]^-$ at *m*/*z* 359.1859 afforded a product ion at *m*/*z* 329.1742 $[M-H-CH_2O]$ at *m*/*z* 285.1781 and at *m*/*z* 283.1695. Compound **20** was identified as epirosmanol methyl ether.
